# Associations of sickness absence for pain in the low back, neck and shoulders with wider propensity to pain

**DOI:** 10.1136/oemed-2019-106193

**Published:** 2020-02-20

**Authors:** David Coggon, Georgia Ntani, Karen Walker-Bone, Vanda E Felli, Raul Harari, Lope H Barrero, Sarah A Felknor, Marianela Rojas, Anna Cattrell, Consol Serra, Matteo Bonzini, Eleni Solidaki, Eda Merisalu, Rima R Habib, Farideh Sadeghian, M Masood Kadir, A Rajitha Wickremasinghe, Ko Matsudaira, Busisiwe Nyantumbu-Mkhize, Helen L Kelsall, Helen Harcombe

**Affiliations:** 1 Medical Research Council Lifecourse Epidemiology Unit, University of Southampton, Southampton, UK; 2 Arthritis Research UK/MRC Centre for Musculoskeletal Health and Work, Univeristy of Southampton, Southampton, UK; 3 School of Nursing, University of São Paulo, São Paulo, Brazil; 4 Corporación para el Desarrollo de la Producción y el Medio Ambiente Laboral – IFA (Institute for the Development of Production and the Work Environment), Quito, Ecuador; 5 Department of Industrial Engineering, School of Engineering, Pontificia Universidad Javeriana, Bogotá, Colombia; 6 Southwest Center for Occupational and Environmental Health, The University of Texas Health Science Center at Houston School of Public Health, Houston, Texas, USA; 7 Center for Disease Control and Prevention/National Institute for Occupational Safety and Health, Atlanta, Georgia, USA; 8 Program Health, Work and Environment in Central America, Institute for Studies on Toxic Substances (IRET), National University of Costa Rica, Heredia, Costa Rica; 9 North East London NHS Foundation Trust, Goodmayes Hospital, Ilford, Essex, UK; 10 Center for Research in Occupational Health (CiSAL), University Pompeu Fabra, Barcelona, Spain; 11 CIBER of Epidemiology and Public Health, Barcelona, Spain; 12 IMIM (Hospital del Mar Research Institute), Barcelona, Spain; 13 Occupational Health Service, Parc de Salut MAR, Barcelona, Spain; 14 Department of Clinical Science and Community Health, University of Milan and Fondazione IRCCS Policlinico, Milano, Italy; 15 Department of Social Medicine, Medical School, University of Crete, Heraklion, Greece; 16 Institute of Technology, Estonian University of Life Sciences, Tartu, Estonia; 17 Department of Environmental Health, Faculty of Health Sciences, American University of Beirut, Beirut, Lebanon; 18 Center for Health Related Social and Behavioral Sciences Research, Shahroud University of Medical Sciences, Shahroud, Iran; 19 Department of Community Health Sciences, Aga Khan University, Karachi, Pakistan; 20 Faculty of Medicine, University of Kelaniya, Ragama, Western, Sri Lanka; 21 Department for Medical Research and Management for Musculoskeletal Pain, 22nd Century Medical and Research Center, Faculty of Medicine, The University of Tokyo Hospital, Tokyo, Japan; 22 National Institute for Occupational Health, National Health Laboratory Service, Johannesburg, South Africa; 23 Faculty of Health Sciences, School of Public Health, University of Witwatersrand, Johannesburg, South Africa; 24 Department of Epidemiology and Preventive Medicine, School of Public Health and Preventive Medicine, Monash University, Melbourne, Victoria, Australia; 25 Department of Preventive and Social Medicine, University of Otago, Dunedin, New Zealand

**Keywords:** epidemiology, musculoskeletal, longitudinal studies

## Abstract

**Objectives:**

To explore the association of sickness absence ascribed to pain at specific anatomical sites with wider propensity to musculoskeletal pain.

**Methods:**

As part of the CUPID (Cultural and Psychosocial Influences on Disability) study, potential risk factors for sickness absence from musculoskeletal pain were determined for 11 922 participants from 45 occupational groups in 18 countries. After approximately 14 months, 9119 (78%) provided follow-up information about sickness in the past month because of musculoskeletal pain, including 8610 who were still in the same job. Associations with absence for pain at specific anatomical sites were assessed by logistic regression and summarised by ORs with 95% CIs.

**Results:**

861 participants (10%) reported absence from work because of musculoskeletal pain during the month before follow-up. After allowance for potential confounders, risk of absence ascribed entirely to low back pain (n=235) increased with the number of anatomical sites other than low back that had been reported as painful in the year before baseline (ORs 1.6 to 1.7 for ≥4 vs 0 painful sites). Similarly, associations with wider propensity to pain were observed for absence attributed entirely to pain in the neck (ORs up to 2.0) and shoulders (ORs up to 3.4).

**Conclusions:**

Sickness absence for pain at specific anatomical sites is importantly associated with wider propensity to pain, the determinants of which extend beyond established risk factors such as somatising tendency and low mood. Better understanding of why some individuals are generally more prone to musculoskeletal pain might point to useful opportunities for prevention.

**Key messages**

**What is already known about this subject?**

- Recent research suggests that wide international variation in the prevalence of self-reported disabling low back and wrist/hand pain among working populations is driven largely by unidentified factors predisposing to musculoskeletal pain in general, rather than by factors specific to the site at which symptoms occur. However, the findings could have occurred because some individuals tend to recall and report pain more readily than others.

**What are the new findings?**

- We demonstrate that sickness absence for pain in the low back, neck and shoulders, reporting of which should be less subjective than that of difficulty with everyday activities, is also importantly associated with wider propensity to pain, as indicated by the extent to which other parts of the body had earlier been reported as painful.

**How might this impact on policy or clinical practice in the foreseeable future?**

- Better understanding of why some individuals and populations are generally more prone to musculoskeletal pain may point to opportunities for prevention of such pain, and thereby of the sickness absence and other disability that it causes.

## Introduction

Using longitudinal data from the Cultural and Psychosocial Influences on Disability (CUPID) study, we have previously shown that after adjustment for other known and suspected risk factors, self-reported disabling pain in the low back and wrist/hand (ie, pain that was reported as making specified everyday activities difficult or impossible) was strongly related to the extent of pain at other anatomical sites, assessed some 14 months earlier.^1 2^ Prevalence rate ratios (PRRs) for disabling low back pain increased progressively from 1.4 to 2.6, as the number of other anatomical sites that had been painful rose from 1 through to ≥6.^1^ For disabling wrist/hand pain, the corresponding gradient in PRRs was from 1.4 to 3.6.^2^ Furthermore, much of the large variation between countries and occupations in the prevalence of disabling pain appeared to be driven by differences in general propensity to the symptom, for which the reported number of sites with pain served as an index.^1 2^ In support of this, baseline prevalence rates of disabling pain in the low back and wrist/hand were highly variable but strongly correlated across the 47 occupational groups that contributed to the study (r=0.76).^3^

This pattern of results could not be a consequence of localised pathology in peripheral tissues. In theory, it might be explained by some frequently occurring, but as yet unidentified, systemic pathology, or by one or more external physical factors that cause pain across the trunk and all limbs. However, it seems unlikely that such causes would have escaped detection in the extensive body of research that has been carried out on regional and multisite musculoskeletal pain. More plausible is the possibility that the findings reflect physiological differences in the processing of sensory information (perhaps psychologically driven) that render some individuals generally more susceptible to pain. If correct, this could have important implications for strategies to prevent disabling musculoskeletal pain in working populations.

It is also possible, however, that the observed associations occurred simply because some individuals, particularly in certain cultural environments, have a generally lower threshold for reporting pain and associated disability, whereas others tend to make light of any symptoms. This type of reporting artefact might be expected to apply less to more definitive measures of disability from pain, such as sickness absence, which should be less prone to subjective differences in reporting.

To address this potential for reporting artefact, we therefore carried out further analysis of data from the CUPID study to explore whether sickness absence ascribed to musculoskeletal pain at specific anatomical sites was also associated with wider propensity to pain as indicated by the extent of pain elsewhere.

## Methods

### Initial study sample

The methods of the CUPID study, including ethical approvals, have been described in detail elsewhere.^4^ During 2006–2011, a baseline questionnaire was completed, either through self-administration or at interview, by 12 426 participants from 47 occupational groups in 18 countries across five continents, with an overall response rate of 70%. The occupational groups fell into three broad categories—nurses, office staff and other workers (most of whom carried out repetitive manual tasks with their hands or arms).

### Baseline questionnaire

The questionnaire was originally drafted in English and then translated into local languages with checks for accuracy by independent back-translation. Among other things, it asked about various demographic, lifestyle, psychological and occupational risk factors for musculoskeletal pain and associated disability (table 1). Questions about mental health were taken from the SF-36 questionnaire,^5^ and scores were grouped to three levels (good, intermediate and poor) corresponding to approximate thirds of their distribution in the full study sample. Questions about distress from common somatic symptoms were derived from the Brief Symptom Inventory^6^ and provided a measure of somatising tendency in the number of symptoms from a total of five (faintness or dizziness, pains in the heart or chest, nausea or upset stomach, trouble getting breath, and hot or cold spells) that were reported as at least moderately distressing in the past week. Questions on beliefs about pain in the low back and upper limb were adapted from the Fear Avoidance Beliefs Questionnaire.^7^ Participants were deemed to have adverse beliefs about the work-relatedness of a pain if they completely agreed that it was commonly caused by work; about its relationship to physical activity if they completely agreed that for someone with the pain, physical activity should be avoided as it might cause harm, and that rest was needed to get better; and about its prognosis if they completely agreed that neglecting such problems could cause serious harm, and completely disagreed that such problems usually got better within 3 months. Questions about physical demands of work were framed in relation to ‘an average working day’. Time pressure at work was defined by report of a target number of articles or tasks to be finished in the day or working under pressure to complete tasks by a fixed time, and incentives by piecework or payment of a bonus if more than an agreed number of articles/tasks were finished in a day. Support at work was deemed to be lacking if the participant said that it was seldom or ever provided by either colleagues or a supervisor/manager.

**Table 1:** Personal risk factors that were analysed

Variable	Classification
**Demographic**	
Sex	Male (n=2935); female (n=5675)
Age at baseline (years)	20–29 (n=1944); 30–39 (n=2777); 40–49 (n=2499); 50–59 (n=1390)
**Lifestyle**	
Smoking	Never (n=5555); ex-smoker (n=1201); current (n=1831); not known (n=23)
**Psychological**	
Mental health	Good (n=3414); intermediate (n=2603); poor (n=2562); not known (n=31)
No of distressing somatic symptoms	0 (n=5138); 1 (n=1894); ≥2 (n=1532); not known (n=46)
Adverse health beliefs about low back pain*	
Work-relatedness	No (n=5628); yes (n=2982)
Physical activity	No (n=6949); yes (n=1661)
Prognosis	No (n=7402); yes (n=1208)
Adverse health beliefs about arm/shoulder/hand pain†‡§	
Work-relatedness	No (n=5989); yes (n=2621)
Physical activity	No (n=7543); yes (n=1067)
Prognosis	No (n=7722); yes (n=888)
**Occupational activities in an average working day**	
Lift weights of 25 kg or more by hand*†‡	No (n=5510); yes (n=3100)
Work for >1 hour in total with hands above shoulder height†‡	No (n=5812); yes (n=2798)
Use of keyboard or typewriter for >4 hours in total†‡§	No (n=5496); yes (n=3114)
Other tasks involving repeated movements of the wrist or fingers for >4 hours in total§	No (n=3230); yes (n=5380)
**Psychosocial aspects of work**	
Work >50 hours per week	No (n=6670); yes (n=1940)
Time pressure	No (n=2181); yes (n=6429)
Incentives	No (n=6230); yes (n=2380)
Lack of support	No (n=6359); yes (n=2251)
Job dissatisfaction	No (n=6950); yes (n=1660)
Lack of control	No (n=6878); yes (n=1732)
Job insecurity	No (n=6074); yes (n=2536)
**Previous sickness absence**	
Absence for >5 days in total in year before baseline for non-musculoskeletal problems	No (n=7841); yes (n=769)
**Pain propensity**	
Pain propensity index¶	0; 1; 2; 3; 4; 5; ≥6 (numbers varied by outcome)

*Analyses of sickness absence for low back pain.

†Analyses of sickness absence for neck pain.

‡Analyses of sickness absence for shoulder pain.

§Analyses of sickness absence for wrist/hand pain.

¶For each outcome, pain propensity index was defined according to the number of anatomical sites, excluding the outcome site, that had been painful in the year before baseline (see text).

A further question asked about the total duration of absence from work in the past year because of non-musculoskeletal health problems (0 days/1–5 days/6–30 days/>30 days), which for this report was classified according to whether or not it exceeded 5 days.

In addition, the baseline questionnaire asked participants whether or not in the past year they had experienced pain lasting at least a day in each of 10 anatomical sites—low back, neck and right and left shoulder(s), elbow(s), wrist/hand(s) and knee(s). Answers to these questions were used to define measures of general propensity to musculoskeletal pain (see below in section on Statistical analysis).

### Group-level risk factors

Also at baseline, the lead investigator in each country provided information about six possible risk factors defined at occupational group level. These were the unemployment rate in the community from which the group was drawn, whether workers were eligible for full pay during the first 3 months of sickness absence, whether there was social security for long-term unemployment, whether financial support was provided in the event of ill-health retirement, whether it was necessary to pay for primary care, and whether compensation was paid for work-related back or arm pain.

### Follow-up

After an interval of approximately 14 months, participants from all but two of the occupational groups (manual workers in Costa Rica and office workers in South Africa) were asked to complete a shorter follow-up questionnaire, again by self-administration or at interview. This included questions about absence from work during the past month because of pain in the low back, neck, shoulder(s), elbow(s), wrist/hand(s) and knee(s).

### Statistical analysis

Statistical analysis was carried out with Stata V.12.1 software (Stata Corp LP 2012; Stata Statistical Software, College Station, Texas, USA). For each of four categories of pain (low back, neck, shoulder(s) and wrist/hand(s)), we derived an index of wider propensity to pain defined by the number of other anatomical sites that had been painful for a day or longer in the year before baseline. Thus, for example, the index for the low back ranged from 0 to 9 and that for the shoulder(s) from 0 to 8.

We also derived 11 further group-level risk factors, using data from the baseline questionnaires completed by individual participants. These were the prevalence by occupational group of six adverse beliefs about musculoskeletal pain, the group prevalence of absence for >5 days in the past year because of non-musculoskeletal health problems, and group mean pain propensity indices specific to each of the four categories of pain (table 2).

**Table 2:** Group-level risk factors that were analysed

Variable	Classification
Unemployment rate >10%	No (n=6875 in 34 occupational groups) Yes (n=1735 in 11 occupational groups)
Full sick pay in first 3 months of absence	No (n=3003 in 21 occupational groups) Yes (n=5607 in 24 occupational groups)
Lack of social security support for long-term unemployment	No (n=5496 in 26 occupational groups) Yes (n=3114 in 19 occupational groups)
Support for ill-health retirement	No (n=4110 in19 occupational groups) Yes (n=4500 in 26 occupational groups)
Payment for primary care	No (n=5803 in 27 occupational groups) Yes (n=2807 in 18 occupational groups)
Compensation for back/arm pain	No (n=1635 in 9 occupational groups) Yes (n=6975 in 36 occupational groups)
Group prevalence of adverse health beliefs about low back pain*	
Work-relatedness	Continuous (n=8610)
Physical activity	Continuous (n=8610)
Prognosis	Continuous (n=8610)
Group prevalence of adverse health beliefs about arm/shoulder/hand pain†	
Work-relatedness	Continuous (n=8610)
Physical activity	Continuous (n=8610)
Prognosis	Continuous (n=8610)
Group prevalence of absence for >5 days in total during year before baseline for non-musculoskeletal problems	Continuous (n=8610)
Group mean pain propensity index‡	Continuous (n=8610)

*Analyses of sickness absence for low back pain.

†Analyses of sickness absence for neck pain, shoulder pain and wrist/hand pain.

‡For each outcome, pain propensity index was defined according to the number of anatomical sites, excluding the outcome site, that had been painful in the year before baseline (see text).

After generating preliminary descriptive statistics, we focused first on two outcomes—sickness absence (of any duration) in the month before follow-up that was ascribed (1) at least in part, and (2) entirely, to pain in the low back. Using logistic regression (with random intercepts for occupational group to allow for the hierarchical structure of the data), we examined their univariate associations with each of the personal risk factors from table 1, retaining those that were significant at a 10% level for either outcome. Next, we examined associations with each of the group-level risk factors in table 2 in separate logistic regression models that adjusted for the personal risk factors retained from the first step (by definition, group-level variables took an identical value for each member of the same occupational group). Again, we retained those that were associated with either outcome at a 10% level of significance. We then fitted final models, one for each of the two outcomes, incorporating all of the risk factors, both personal and group-level, that had been retained from the earlier analyses. Associations were summarised by ORs with 95% CIs. For the outcome of sickness absence attributed entirely to low back pain, we also carried out supplementary analyses using the same explanatory variables, but stratified according to whether or not low back pain had been reported in the year before baseline.

Similar analyses were then performed for sickness absence attributed to pain in the neck, shoulder(s) and wrist/hand(s).

## Results

Among the 45 occupational groups that contributed to the longitudinal component of the CUPID study, 11 992 participants answered the baseline questionnaire, including 11 702 who provided usable information on musculoskeletal pain during the year before baseline. Of those, 9119 (78%) completed follow-up, but 509 were excluded from further analysis because they had changed their job since baseline. This left a final sample of 8610 participants on which the analyses for this report were based. Tables 1 and 2 summarise the distribution of risk factors across the study sample.

In total, 861 participants (10%) reported absence from work during the month before follow-up because of musculoskeletal pain. In most cases (560), the pain was limited to only one of low back, neck, shoulder(s), elbow(s), wrist/hand(s) or knee(s), but a substantial minority (301) ascribed their absence to pain in two or more regions.

Absence due at least in part to pain in the low back was reported by 439 participants, including 235 in whom it was given as the only reason. Table 3 shows the risk factors that were significantly associated (p<0.05) with these outcomes in the final regression models. Absence ascribed at least in part to low back pain (LBP) was associated with somatising tendency (OR 1.7), absence in the year before baseline for non-musculoskeletal reasons (OR 1.3), lack of social security support for long-term unemployment (OR 1.8), lower group prevalence of adverse beliefs about the prognosis of LBP (OR 0.7 for an increase in prevalence of one SD) and higher group prevalence of absence for >5 days in the past year for non-musculoskeletal health problems (OR 1.3 for an increase in prevalence of one SD). In addition, after allowance for these and other potential confounders, it was strongly associated with baseline report of pain at other anatomical sites (ORs 1.5 to 2.3). When attention was restricted to absence attributed entirely to LBP, the association with pain elsewhere was reduced a little (ORs 1.1 to 1.7), but remained significant at a 5% level. Stratification of that analysis indicated that the association with pain at other anatomical sites was limited to participants who had not reported LBP in the year before baseline (online supplementary table 1).

10.1136/oemed-2019-106193.supp1 Supplementary data

**Table 3:** Statistically significant baseline risk factors for sickness absence attributed to low back pain in month before follow-up

Risk factor	No sickness absence for low back pain	Absence attributed all or in part to low back pain			Absence attributed only to low back pain		
		N	*OR	(95% CI)	N	*OR	(95% CI)
No of distressing somatic symptoms in past week							
0	4966	172	1		105	1	
1	1778	116	1.4	(1.1 to 1.8)	62	1.4	(1.0 to 2.0)
≥2	1382	150	1.7	(1.3 to 2.2)	68	1.7	(1.2 to 2.4)
Absence in past year for non-musculoskeletal health problems							
>5 days	710	59	1.3	(1.0 to 1.8)	36	1.5	(1.0 to 2.2)
Factors defined at occupational group level							
Prevalence of adverse beliefs about prognosis of low back pain (1 SD increase)			0.7	(0.6 to 1.0)		0.8	(0.7 to 0.9)
Prevalence of absence (>5 days) in past year for non-musculoskeletal health problems (1 SD increase)			1.3	(1.0 to 1.7)		1.3	(1.1 to 1.6)
Lack of social security support for long-term unemployment			1.8	(1.1 to 3.1)		1.7	(1.2 to 2.5)
Pain propensity index							
0	2490	71	1		53	1	
1	1786	88	1.5	(1.1 to 2.2)	45	1.2	(0.8 to 1.8)
2	1401	74	1.5	(1.0 to 2.1)	42	1.3	(0.8 to 1.9)
3	1053	71	1.7	(1.2 to 2.5)	28	1.1	(0.7 to 1.8)
4	619	46	1.6	(1.1 to 2.5)	26	1.6	(1.0 to 2.7)
5	420	35	1.6	(1.0 to 2.5)	20	1.7	(0.9 to 3.0)
≥6	402	54	2.3	(1.5 to 3.6)	21	1.6	(0.9 to 2.9)

*ORs with 95% CIs derived from a single logistic regression model for each of the two outcomes that included all of the risk factors listed together with sex, age (four strata), mental health, personal adverse beliefs about low back pain (work-relatedness, prognosis), lack of support at work, time pressure at work, job dissatisfaction, availability of compensation for low back pain, group prevalence of adverse beliefs about low back pain and physical activity, and payment for primary care. Risk estimates are presented only for factors that were significantly associated (p<0.05) with at least one of the two outcomes.

A total of 302 participants reported absence at least in part because of neck pain, which was significantly associated (p<0.05) with somatising tendency, job dissatisfaction, adverse personal beliefs about prognosis, group prevalence of sickness absence in the year before baseline for non-musculoskeletal reasons, group prevalence of adverse health beliefs about the work-relatedness of arm pain, baseline report of pain at other sites (ORs 1.3 to 2.7) and group mean pain propensity index (OR for an increase of one SD 1.7, 95% CI 1.3 to 2.4) (table 4). When attention was restricted to the 97 subjects who gave neck pain as the only reason for their absence, most associations were attenuated, but that with individual report of pain at other sites remained significant at a 5% level (ORs up to 2.0). In stratified analyses, the association between absence attributed entirely to neck pain and the extent of pain at other anatomical sites was stronger among participants who reported neck pain in the year before baseline (online supplementary table 2).

**Table 4:** Statistically significant baseline risk factors for sickness absence attributed to neck pain in month before follow-up

Risk factor	No sickness absence for neck pain	Absence attributed all or in part to neck pain			Absence attributed only to neck pain		
		N	*OR	(95% CI)	N	*OR	(95% CI)
Smoking habits							
Never	5331	224	1		71	1	
Ex-smoker	1171	30	0.8	(0.5 to 1.2)	6	0.4	(0.2 to 0.9)
Current	1784	47	1.1	(0.8 to 1.6)	20	1.1	(0.6 to 1.9)
No of distressing somatic symptoms in past week							
0	5022	116	1		44		
1	1825	69	1.1	(0.8 to 1.5)	24	1.2	(0.7 to 2.0)
≥2	1419	113	1.6	(1.2 to 2.2)	27	1.6	(0.9 to 2.7)
Psychosocial aspects of work							
Job dissatisfaction	1597	63	1.5	(1.0 to 2.0)	18	1.1	(0.6 to 1.9)
Adverse health beliefs about arm pain							
Poor prognosis	839	49	1.4	(1.0 to 2.0)	15	1.4	(0.8 to 2.5)
Factors defined at occupational group level							
Prevalence of absence (>5 days) in past year for non-musculoskeletal health problems (1 SD increase)			1.3	(1.0 to 1.6)		1.4	(1.1 to 1.9)
Group mean pain propensity index (1 SD increase)			1.7	(1.3 to 2.4)		1.4	(1.0 to 1.8)
Prevalence of adverse health beliefs about arm pain (work-relatedness) (1 SD increase)			0.7	(0.5 to 0.9)		0.7	(0.5 to 0.9)
Payment for primary care			1.8	(1.1 to 3.0)		1.2	(0.7 to 2.0)
Pain propensity index							
0	2143	32	1		13	1	
1	2033	49	1.3	(0.8 to 2.0)	21	1.5	(0.7 to 3.0)
2	1448	57	1.8	(1.1 to 2.9)	21	1.9	(0.9 to 3.8)
3	1127	60	2.1	(1.3 to 3.4)	19	2.0	(1.0 to 4.2)
4	668	34	1.8	(1.0 to 3.0)	12	1.9	(0.8 to 4.3)
5	465	27	1.6	(0.9 to 2.8)	6	1.2	(0.4 to 3.5)
≥6	424	43	2.7	(1.6 to 4.5)	5	1.0	(0.3 to 3.0)

*ORs with 95% CIs derived from a single logistic regression model for each outcome that included all of the risk factors listed together with sex, mental health, personal absence from work in past year for non-musculoskeletal health problems, personal adverse beliefs about the work-relatedness of arm pain, lack of support at work and job insecurity. Risk estimates are presented only for factors that were significantly associated (p<0.05) with at least one of the two outcomes.

Findings from the final models for shoulder pain are summarised in table 5, and are based on 214 cases in whom absence was ascribed at least partially to such pain and 57 in whom no other reason for the absence was given. Somatising tendency and report of pain at other sites were risk factors for both outcomes with ORs for the latter up to 3.8 and 3.4. After stratification, the association of absence for pain only in the shoulder(s) with pain at other anatomical sites was most clearly apparent among subjects with shoulder pain in the year before baseline (online supplementary table 3).

**Table 5:** Statistically significant baseline risk factors for sickness absence attributed to shoulder pain in month before follow-up

Risk factor	No sickness absence for shoulder pain	Absence attributed all or in part to shoulder pain			Absence attributed only to shoulder pain		
		N	*OR	(95% CI)	N	*OR	(95% CI)
Smoking habits							
Never	5391	164	1		50	1	
Ex-smoker	1175	26	1.1	(0.7 to 1.7)	3	0.3	(0.1 to 1.0)
Current	1807	24	0.8	(0.5 to 1.2)	4	0.3	(0.1 to 0.8)
No of distressing somatic symptoms in past week							
0	5059	79	1		28	1	
1	1840	54	1.3	(0.9 to 1.8)	13	1.2	(0.6 to 2.3)
≥2	1452	80	1.8	(1.2 to 2.5)	16	1.6	(0.8 to 3.3)
Adverse health beliefs about arm pain							
Need to avoid physical activity	1032	35	1.6	(1.1 to 2.4)	5	0.8	(0.3 to 2.2)
Pain propensity index							
0	1948	20	1		7	1	
1	1983	34	1.5	(0.9 to 2.7)	16	2.4	(0.9 to 5.8)
2	1787	40	1.8	(1.0 to 3.1)	11	1.7	(0.6 to 4.6)
3	1165	46	2.6	(1.5 to 4.5)	10	2.3	(0.8 to 6.3)
4	749	27	2.1	(1.1 to 3.9)	4	1.5	(0.4 to 5.4)
5	394	18	2.3	(1.1 to 4.6)	4	2.7	(0.7 to 10.0)
≥6	370	29	3.8	(2.0 to 7.2)	5	3.4	(1.0 to 12.2)

*ORs with 95% CIs derived from a single logistic regression model for each outcome that included all of the risk factors listed together with age (four strata), mental health, personal adverse beliefs about arm pain (poor prognosis), work with hands above shoulder height for >1 hour per day, work for >50 hours per week, group prevalence of adverse beliefs about need to avoid physical activity with arm pain and group mean pain propensity index. Risk estimates are presented only for factors that were significantly associated (p<0.05) with at least one of the two outcomes.

Fewer cases of absence were ascribed to wrist/hand pain (147 overall), and fewer risk factors showed significant associations with the outcome. However, they again included report of pain at other sites (ORs 1.2 to 3.5). Only 50 participants attributed absence exclusively to wrist/hand pain, and associations with pain elsewhere were less clear, although ORs tended to be elevated when baseline pain at other sites was most extensive (online supplementary table 4).

## Discussion

This longitudinal analysis built on earlier work which suggested that wide international variation in the prevalence of disabling musculoskeletal pain among working populations is importantly driven by one or more risk factors that predispose to musculoskeletal pain in general.^1 2^ It showed that previously demonstrated associations with pain propensity extended to recall of recent sickness absence for musculoskeletal pain, chosen for study because it was a less subjective outcome than self-report of difficulty with everyday activities. This indicates that the earlier findings were not simply a reporting artefact, and is further encouragement to explore why some individuals and populations are generally more prone to musculoskeletal pain.

Our investigation had the advantage of a large and diverse study sample with good response rates at follow-up. The measures of general propensity to pain that it employed were the same as, or analogous to, those in the earlier research on which it built.^1 2^ They were intended as indices of exposure to one or more as yet unidentified factors that predispose to musculoskeletal pain in general, and for that purpose it was not necessary that the pain at different sites should occur simultaneously or close in time (as is usually required in studies of multisite or widespread pain). It was important, however, to exclude the outcome anatomical site from each measure. Otherwise, associations might in part reflect the well-established tendency for musculoskeletal pain at a given site to be persistent and recurrent.^8 9^

The longitudinal design meant that the ascertainment of risk factors, including the extent of pain at other anatomical sites, preceded and could not be influenced by the outcomes under investigation. Thus, while recall of some exposures may not have been completely accurate, any errors are generally likely to have been non-differential with respect to the outcomes, and as such would tend to bias risk estimates towards the null. A possible exception is sickness absence in the year before baseline for non-musculoskeletal reasons. If some individuals tended systematically to under-report all types of sickness absence, risk estimates for that measure could have been biased in either direction. We would, however, expect any such effect to be small since sickness absence in the past month (the outcome) is a relatively memorable event and should have been assessed fairly reliably.

While recall of recent sickness absence for musculoskeletal pain was a less subjective outcome than report of pain causing disability for everyday activities, it was also less frequent, which tended to reduce the precision of risk estimates, and may explain why exposure–response relationships were less consistent than in earlier analyses with disability for everyday activities as the outcome.^1 2^ Moreover, it was a less direct marker of disabling pain, potentially being influenced also by other factors such as sickness absence behaviour and culture, and the scope for temporary redeployment when symptoms occurred. We attempted to control where necessary for confounding by such factors, as well as by other known determinants of musculoskeletal pain such as somatising tendency. In addition, the inclusion of random intercepts for occupational group in our regression models should have reduced any residual confounding by risk factors acting at group level on which we did not have information, as well as addressing spurious precision from clustering effects.

Importantly, our analyses did not adjust for earlier report of pain at the index site. Such pain would lie on the causal pathway between the hypothesised unidentified causes of general propensity to pain and the sickness absence outcome, and therefore would not be a confounder. We did, however, carry out supplementary analyses stratified according to whether pain at the index site had been reported in the year before baseline (online supplementary tables 1–3). These subanalyses were subject to greater statistical uncertainty, and the absence of pain at a site in the year before baseline does not preclude its having been present longer in the past. However, the findings for neck and shoulder pain suggest that part, at least, of the impact of general propensity to pain is on the persistence and/or recurrence of symptoms.

Within the study sample, the overall 1-month prevalence of sickness absence at follow-up because of musculoskeletal pain was 10%. In most cases, the absence was attributed to pain at a single anatomical site, but a substantial minority reported contributions from pain in several bodily regions. In these circumstances, the observed associations with pain propensity may in part have reflected the persistence or recurrence of pain at one or more sites. However, associations were apparent even when absence for pain at multiple sites was excluded, and as already mentioned, we took care to exclude the outcome site of pain when deriving our measures of pain propensity.

Because a worker’s sickness absence history is an important predictor of future sickness absence episodes,^10–12^ we included earlier sickness absence for non-musculoskeletal health problems as a potential risk factor in our analyses. As expected, past sickness absence was significantly associated with absence for LBP, while absence for neck pain was associated with the group prevalence of absence for non-musculoskeletal health problems. However, these risk factors did not explain the associations with pain propensity index when they were included in the regression models.

Of the other potentially confounding variables that were associated with absence ascribed to musculoskeletal pain, somatising tendency was the most consistent, showing associations for pain in each of the low back, neck and shoulder. A relationship of somatising tendency to sickness absence has been reported before,^13^ and it is plausible that heightened perception of, and anxiety about, symptoms could contribute to an individual’s ability to cope at work and decisions to take sickness absence. Again, however, adjustment for somatising tendency did not eliminate associations with pain propensity.

It is highly plausible that sickness absence attributed to pain at one anatomical site should be associated with earlier pain elsewhere, given the tendency for musculoskeletal pain often to occur at multiple sites.^14–17^ Moreover, LBP has been shown by several investigators to be predicted by pain elsewhere,^18 19^ and the new findings presented here on sickness absence are consistent with our earlier publications in which we showed that pain at other anatomical sites was associated with subsequent report of disability for everyday activities because of pain in the low back^1^ and wrist/hand.^2^ Importantly, they indicate that those associations with report of disability did not arise simply because some individuals have a lower threshold than others for reporting pain and disability, and they thus add weight to the evidence that major international differences in the prevalence of disabling musculoskeletal pain among working populations are importantly driven by causes that predispose to musculoskeletal pain in general and not just in localised anatomical regions.

In summary, our results suggest that the previously reported associations of self-reported disabling musculoskeletal pain with earlier complaint of pain at other anatomical sites are not simply a consequence of subjective differences in thresholds for reporting symptoms and disability. They suggest that across a diverse range of countries, general propensity to musculoskeletal pain is an important determinant of pain at specific anatomical sites, and thereby of associated disability, including sickness absence. They thus reinforce the need to understand better what drives such propensity and ultimately to find ways in which it might be reduced.
