# Supplementary material for: Associations of sickness absence for pain in the low back, neck and shoulders with wider propensity to pain
Source: Occup Environ Med. 2020 Feb 20;77(5):301–8. doi: 10.1136/oemed-2019-106193 (PMC7231440; doi:10.1136/oemed-2019-106193)
Supplement: Supplementary data [file oemed-2019-106193supp001.pdf]

Supplementary Table 1 Association of pain propensity index with sickness absence in month before follow-up attributed only to low back pain, according to report of low back pain in the 12 months before baseline

Risk factor	Low back pain in 12 months before baseline				No low back pain in 12 months before baseline			
	Sickness absence		[a]OR	(95% CI)	Sickness absence		[a]OR	(95% CI)
	No	Yes			No	Yes		
**Pain propensity index**								
0	753	37	1		1,737	16	1	
1	956	35	0.7	(0.5,1.2)	830	10	1.4	(0.6,3.2)
2	836	29	0.6	(0.4,1.0)	565	13	2.5	(1.2,5.6)
3	740	25	0.6	(0.4,1.1)	313	3	1.1	(0.3,4.0)
4	483	24	0.8	(0.5,1.4)	136	2	1.5	(0.3,6.9)
5	342	17	0.8	(0.4,1.4)	78	3	4.3	(1.1,16.6)
≥6	364	20	0.7	(0.4,1.3)	38	1	2.6	(0.3,21.8)

[a]Odds ratios with 95% confidence intervals derived from a logistic regression model with adjustment for all of the risk factors listed in Table 3

Supplementary Table 2 Association of pain propensity index with sickness absence in month before follow-up attributed only to neck pain, according to report of neck pain in the 12 months before baseline

Risk factor	Neck pain in 12 months before baseline				No neck pain in 12 months before baseline			
	Sickness absence		[a]OR	(95% CI)	Sickness absence		[a]OR	(95% CI)
	No	Yes			No	Yes		
**Pain propensity index**								
0	400	3	1		1,743	10	1	
1	812	9	1.4	(0.4,5.1)	1,221	12	1.5	(0.6,3.5)
2	709	14	2.3	(0.6,8.1)	739	7	1.2	(0.5,3.4)
3	669	14	2.3	(0.6,8.2)	458	5	1.3	(0.4,4.0)
4	449	10	2.3	(0.6,8.9)	219	2	0.9	(0.2,4.3)
5	340	5	1.4	(0.3,6.1)	125	1	0.9	(0.1,7.2)
≥6	358	3	0.7	(0.1,3.8)	66	2	2.6	(0.5,15.2)

[a]Odds ratios with 95% confidence intervals derived from a logistic regression model with adjustment for all of the risk factors listed in Table 4

Supplementary Table 3 Association of pain propensity index with sickness absence in month before follow-up attributed only to shoulder pain, according to report of shoulder pain in the 12 months before baseline

Risk factor	Shoulder pain in 12 months before baseline				No shoulder pain in 12 months before baseline			
	Sickness absence				Sickness absence			
	No	Yes	[a]OR	(95% CI)	No	Yes	[a]OR	(95% CI)
**Pain propensity index**								
0	202	1	1		1,746	6	1	
1	507	4	1.7	(0.2,16.1)	1,476	12	2.2	(0.8,6.0)
2	643	6	2.3	(0.3,19.6)	1,144	5	1	(0.3,3.6)
3	533	7	3.1	(0.4,27.0)	632	3	1.1	(0.3,4.6)
4	417	2	1.1	(0.1,13.4)	332	2	1.4	(0.3,7.6)
5	250	4	4.3	(0.4,41.8)	144	0	-	-
≥6	282	4	3.6	(0.4,36.7)	88	1	3.1	(0.3,28.5)

[a]Odds ratios with 95% confidence intervals derived from a logistic regression model with adjustment for all of the risk factors listed in Table 5

Supplementary Table 4 Statistically significant baseline risk factors for sickness absence attributed to wrist/hand pain in month before follow-up

Risk factor	No sickness absence for low back pain	Absence attributed all or in part to low back pain			Absence attributed only to low back pain		
		N	[a]OR	(95% CI)	N	[a]OR	(95% CI)
Work for >50 hours per week	1,928	12	0.4	(0.2,0.9)	6	0.5	(0.2,1.2)
**Adverse health beliefs about arm pain**							
Need to avoid physical activity	1,039	28	1.9	(1.2,3.0)	6	1.0	(0.4,2.3)
Poor prognosis	862	26	1.9	(1.2,3.0)	10	2.1	(1.0,4.3)
**Pain propensity index**							
0	1,897	16	1		8	1	
1	1,882	29	1.6	(0.9,3.1)	11	1.3	(0.5,3.3)
2	1,758	22	1.2	(0.6,2.3)	8	0.9	(0.3,2.5)
3	1,215	23	1.5	(0.8,3.0)	6	0.9	(0.3,2.8)
4	866	20	1.7	(0.8,3.4)	8	1.6	(0.6,4.5)
5	458	17	2.4	(1.1,5.0)	4	1.5	(0.4,5.2)
≥6	387	20	3.5	(1.7,7.3)	5	2.0	(0.6,6.7)

[a]Odds ratios with 95% confidence intervals derived from a single logistic regression model for each outcome that included all of the risk factors listed together with sex, mental health, number of distressing somatic symptoms in the past week, and time pressure at work. Risk estimates are presented only for factors that were significantly associated ($p < 0.05$) with at least one of the two outcomes.
